# Common step-wise interventions improved primary care clinic visits and reduced emergency department discharge failures: a large-scale retrospective observational study

**DOI:** 10.1186/s12913-019-4300-1

**Published:** 2019-07-04

**Authors:** Chet D. Schrader, Richard D. Robinson, Somer Blair, Sajid Shaikh, Amy F. Ho, James P. D’Etienne, Jessica J. Kirby, Radhika Cheeti, Nestor R. Zenarosa, Hao Wang

**Affiliations:** 1 Department of Emergency Medicine, Integrative Emergency Services, John Peter Smith Health Network, 1500 S. Main St, Fort Worth, TX 76104 USA; 2 Department of Medical Education, UNTHSC, Fort Worth, TX 76104 USA; 3 Office of Clinical Research, John Peter Smith Health Network, 1500 S. Main St, Fort Worth, TX 76104 USA; 4 Department of Information Technology, John Peter Smith Health Network, 1500 S. Main St, Fort Worth, TX 76104 USA

**Keywords:** Emergency department, Discharge failure, Clinical compliance, Interventions

## Abstract

**Background:**

It is critical to understand whether providing health insurance coverage, assigning a dedicated Primary Care Physician (PCP), and arranging timely post-Emergency Department (ED) clinic follow-up can improve compliance with clinic visits and reduce ED discharge failures. We aim to determine the benefits of providing these common step-wise interventions and further investigate the necessity of urgent PCP referrals on behalf of ED discharged patients.

**Methods:**

This is a single-center retrospective observational study. All patients discharged from the ED over the period Jan 1, 2015 through Dec 31, 2017 were included in the study population. Step-wise interventions included providing charity health insurance, assigning a dedicated PCP, and providing ED follow-up clinics. PCP clinic compliance and ED discharge failures were measured and compared among groups receiving different interventions.

**Result:**

A total of 227,627 patients were included. Fifty-eight percent of patients receiving charity insurance had PCP visits in comparison to 23% of patients without charity insurance (*p* < 0.001). Seventy-seven percent of patients with charity insurance and PCP assignments completed post-ED discharge PCP visits in comparison to only 4.5% of those with neither charity insurance nor PCP assignments (*p* < 0.001).

**Conclusions:**

Step-wise interventions increased patient clinic follow-up compliance while simultaneously reducing ED discharge failures. Such interventions might benefit communities with similar patient populations.

**Electronic supplementary material:**

The online version of this article (10.1186/s12913-019-4300-1) contains supplementary material, which is available to authorized users.

## Background

Traditional practice recommends arranging timely clinic follow-up for patients who are discharged from the Emergency Department (ED). Timely post-ED discharge follow-up has been shown to improve patient-centered care for disease prevention, monitoring, and management [1, 2]. ED discharge failure is defined as patients discharged from ED that either have no primary care physician (PCP) clinic follow-up or that return to the ED inappropriately prior to their clinic visits. However, ED discharge failures are frequent. Nearly one-third of ED patients who do follow up with their primary care physician (PCP) or specialist still have short-term ED returns, and many ED discharged patients may never follow-up with PCP clinics at all [2].

In recently years, numerous studies have been published on transition care from ED to PCP. Studies on utilizing telephone reminders for patient follow-up appointments showed different call success rates with variable outcomes on ED discharge failures [3, 4]. Other studies on a dedicated PCP’s office contacting patients after ED discharge showed increased PCP visits but not decreased ED discharge failures [5, 6]. Among all current interventions, having health insurance and having a follow-up appointment set prior to a patient’s ED departure are considered the most effective for patient follow-up compliance [7].

Many factors could affect patient follow-up after ED discharge [8–10]. Patients with high psychosocial risks or homeless patients might have lower PCP follow-up rates [11]. Patients who lack insurance are reported to have relatively poor PCP follow-up compliance [9, 12]. Patients without a dedicated PCP are also reported to have relatively poor follow-up visit compliance [12, 13]. Realizing these potential contributing factors, different combinations of the following interventions were implemented to improve follow up compliance: providing charity insurance, assigning a dedicated PCP, and providing a phone call or text message to remind patients of post-ED discharge clinic appointments [11, 13, 14]. We assumed that increased patient clinic follow-up compliance would eventually decrease inappropriate ED utilization and/or returns. However, the value of these interventions is varied when analyzing the literature. This variation is due to different interventions rendered in different studies without use of standard outcome measurements [2, 10, 14, 15], thereby requiring further external validation.

The study hospital ED initiated these common interventions together in a step-wise manner. Upon patient discharge, case managers assessed the patient’s qualification for hospital-based charity insurance. If they were able to qualify, steps initiating the charity insurance coverage for patients were begun. Once patients qualified for hospital-based charity insurance, a dedicated PCP was assigned to each patient, and a formal follow-up PCP visit was arranged. In addition, a telephone call to remind the patient of their appointment was made prior to the patient’s PCP visit. The overarching goal was to implement an effective package of interventions to maximize PCP clinic compliance and minimize ED discharge failures. However, commonly, it is challenging to assign dedicated PCPs without insurance coverage or to set up regular PCP visits without dedicated PCPs. Therefore, the study hospital follows a common pragmatic practice by implementing step-wise interventions. At present, we are uncertain of any benefit brought from these step-wise interventions and unable to determine the optimal intervention that eventually could increase clinic compliance and decrease ED discharge failures. Therefore, we aim to investigate the outcomes of providing step-wise interventions and further optimize such interventions for ED discharge failure prevention.

## Methods

### Study setting and design

This was a single-center retrospective study. The study hospital is a publicly funded county hospital and an urban tertiary referral center. The study hospital ED is a level 1 trauma center, acute chest pain and comprehensive stroke center whose annual ED volume was approximately 120,000 visits during the study period (Jan 1, 2015 through Dec 31, 2017). The study ED also sponsors an Emergency Medicine (EM) residency program. This study was approved by the John Peter Smith Health Network Institutional Review Board (IRB number: 010713.004ex). A waived inform consent was granted due to retrospective chart review with no more than minimal risk to subjects.

### Study participants

Patients who presented to the study ED from Jan 1, 2015 through Dec 31, 2017 and were subsequently discharged after the index ED visit were included in this study. All enrolled patients were followed-up by reviewing their Electronic Medical Record (EMR) until Feb 1, 2018. This allowed all enrolled patients a minimum 30-day follow-up after the index ED discharge. We excluded patients during the index ED visits who were 1) admitted, 2) expired, 3) transferred to other facilities, 4) left without being seen (LWBS), eloped, or left against medical advice (AMA), and 5) prisoners.

### Step-wise interventions

Three interventions were already placed and performed in the study hospital ED before the initiation of this study. Therefore, this study can only provide a cross-sectional cohort comparison instead of a pre- and post- intervention comparison. Briefly, patients who had no insurance and who met the criteria listed below were eligible for hospital-based charity insurance coverage. This hospital-based charity insurance coverage is funded by the Texas State Government in United States (US) which lasted for 1 year. It is intended to be a short-term coverage and its use acts as a bridge for patients waiting to be qualified for Medicare, Medicaid, or any commercial insurance coverage . Patients approved for this insurance will need to be reevaluated annually to determine their continuous qualification. Conditional for charity insurance coverage, patients were required to provide no or minimal payment while seeking their healthcare services within the study hospital system. Enrollment eligibility criteria were: 1) patient must be a resident of the local county where hospital is located (Tarrant county); 2) the annual income of the patient or family is below 250% of the federal poverty income level (FPIL); 3) patient is a US citizen, naturalized citizen, or legal permanent resident; and 4) patient has pursued all available health insurance options prior to receiving hospital-based charity insurance coverage. Detailed qualification criteria are on the hospital website (https://www.jpshealthnet.org/for_patients/low_cost_medical_care/do_i_qualify). Only patients who obtained hospital charity insurance were qualified to be assigned a dedicated PCP. A dedicated PCP was assigned randomly based on the availability of physicians taking new patients and their available schedules. All assigned PCPs are within the hospital network system. Once a dedicated PCP was assigned, subsequent formal follow-up clinic appointments could be arranged within 1–4 months of the index ED visits. Patients were informed of PCP assignment and follow-up appointment specifics via mail (US Postal Service). A case manager performed a phone survey to address any potential patient related concerns and to remind patients of their appointments. If no one answered, a message was left to remind patients of their upcoming appointments. Patients were also encouraged to approach hospital/ED social workers or case managers if they had specific questions or concerns. All case managers in the study hospital are licensed master social workers (LMSW) with a Master’s degree in Social Work.

### Outcome measurements

Patient PCP clinic follow-up compliance and ED discharge failure rates were measured as the outcomes. Patient follow-up compliance was defined as patients who visited the PCP clinic after the index ED discharge regardless of ED revisits/returns. In general, discharge failure was defined as ED revisits within a short period of time after the index ED discharge (e.g., 3 days, 7 days, 14 days, or 30 days) and/or poor patient adherence to scheduled PCP clinic follow-up. We defined patients as having no ED discharge failure if they completed a PCP clinic follow-up either before their next appropriate ED revisit or had no further ED revisits. Appropriate ED visits were determined by New York University Algorithm (NYUA), addressed in detail below. We further categorized ED discharge failure as either restricted or broad/uncertain circumstance mediated. Restricted discharge failure was considered if patients met all of the following criteria: 1) patients who returned to the ED prior to their scheduled clinic follow-up visit or returned to the ED with no scheduled clinic follow-up since the index ED visit, and 2) patients who returned to the ED, were discharged and the reasons for ED return were determined inappropriate ED utilization (i.e., non-emergent, avoidable) by NYUA. Broad/uncertain discharge failure excluded patients with restricted discharge failure that met at least one of the following criteria: 1) patients who had neither subsequent ED nor clinic visits; 2) patients who had appropriate or unclassified ED returns but did not have any PCP follow-ups from the index ED discharge; 3) patients that returned to the ED appropriately or unclassified prior to their clinic follow-ups; or 4) patients that returned to the ED after PCP visits and their ED returns were inappropriate or unclassified (A detail definition of different outcomes are listed in Additional file 1).

### Appropriateness of ED utilization

The New York University ED algorithm (NYUA) was used in this study to determine appropriateness of ED utilization for ED return visits [16]. Briefly, four major categories were used in NYUA: 1) emergent not avoidable considered as appropriate ED visits; 2) primary care treatable defined as care that can be safely provided in a primary care setting without the need for emergent treatment; 3) emergent care needed but preventable/avoidable defined as patients whose disease conditions can be prevented/avoided if preventive care is received in a timely fashion; and 4) non-emergent. Patient visits not meeting one of these four major categories were deemed unclassified. Appropriate ED utilization was considered if patients met the emergent not avoidable category criteria, and inappropriate utilization was determined if patients were classified within the other three categories. Uncertain ED utilization was considered if patients were unclassified. NYUA is only used to determine the appropriateness of ED utilization among ED discharged patients. In addition to NYUA, patients who revisited the ED within 30-days were considered appropriate ED utilization where patients were: 1) admitted to hospital, 2) moved to the operating room, 3) transferred to other facilities, or 4) expired.

### Variables

Patient general characteristics including patient age, gender, and race/ethnicity were collected in this study. Other patient and clinical variables were listed as follows: 1) mode of arrival: divided into two categories (healthcare-assisted arrival including ambulance or hospital/healthcare facility-arranged transportation versus others including private car, public transportation, taxi, wheelchair, ambulatory, police, or unknown), 2) level of acuity: divided into two categories based on ESI (Emergency Severity Index) level including high-to-moderate (ESI 1–2-3) and low (ESI 4–5) acuities, 3) homeless status, 4) patient last vital signs upon disposition (including heart rate, respiratory rate, blood pressure, oxygenation, and temperature) divided into two categories of patients who had normal vital signs versus patients with any abnormal vital signs (e.g., heart rate < 50 or > 100, respiratory rate < 8 or > 20, systolic blood pressure < 90 mmHg or > 140 mmHg, diastolic blood pressure < 60 mmHg or > 90 mmHg, pulse oximetry < 94%, temperature > 100.4F° or < 96.8F°), 5) immediate subsequent healthcare visits (e.g., ED, PCP clinic, or none) and its time interval from the index ED discharge, 6) whether patients had their PCP assigned, 7) number of medications prescribed upon the index ED discharge, 8) insurance type divided into five categories including a) hospital-based charity insurance; b) Medicare; c) Medicaid; d) commercial; and e) no insurance coverage; and 9) patient’s chronic disease conditions: divided into two categories including patients with chronic disease(s) and those without. Chronic disease conditions were determined using the chronic condition indicator (CCI) for the International Classification of Diseases Tenth Edition, Clinical Modification (ICD-10-CM). It was developed as part of the Healthcare Cost and Utilization Project (HCUP) by the Agency for Healthcare Research and Quality (AHRQ) [17].

### Study protocol

To determine whether interventions improved patient PCP clinic follow-up compliance, we measured patient PCP clinic follow-up after interventions and compared patients relative to five different insurance coverages (e.g., charity versus Medicare versus Medicaid versus commercial versus no insurance). Furthermore, among patients who complied with their PCP visits, we divided patients into five groups based on differences of their insurance coverage along with three different discharge failure outcomes (e.g., no discharge failure, restricted discharge failure, and broad/uncertain discharge failure). Lastly, the association between the urgency of PCP visits (within 3 days, 7 days, 14 days, and 30 days) and restricted discharge failures were drawn and compared across cohorts of patients with different insurance statuses.

### Data analysis

Student’s t-test was used to compare continuous variables while Fisher’s exact analysis was used to compare categorical variables among groups. A multivariate logistic regression was analyzed to predict clinical compliance and ED restricted discharge failure after patient age, gender, homeless condition, chronic disease condition, mode of arrival at ED, medication prescription, abnormal vital signs upon discharge, and patient level of acuity were adjusted. Besides analyzing three interventions as potential individual independent variables, we also analyzed all three intervention interactions to best fit for pragmatic step-wise implementation. All descriptive and statistical analyses were conducted using STATA 14.2 (College Station, TX). A *p* value < 0.05 was considered statistically significant.

## Results

### General information

**Fig. 1:**
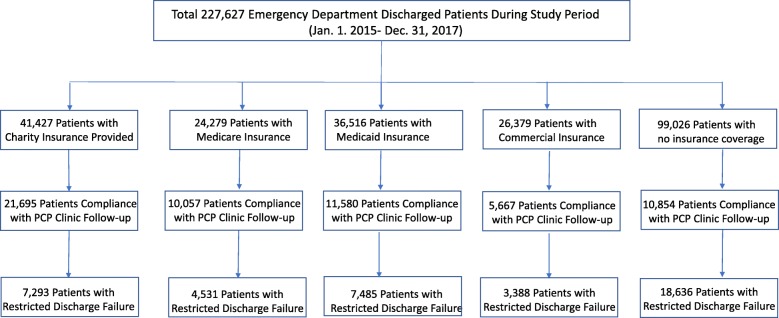
Study Flow Diagram

A total of 227,627 ED discharged patients were retrieved from the EMR. Of these, 41,427 (18%) patients received hospital-based charity insurance coverage with 21,695 (10%) patients eventually following up through a PCP clinic compared to 7293 (3%) patients experiencing restricted discharge failures (Fig. 1). Overall, 30% (67,674/227,627) of patients had at least one clinic follow-up visit after the index ED discharge, 18% (41,333/227,627) of patients were considered restricted discharge failures and only 10% (23,322/227,627) of ED discharged patients had no discharge failure. Table 1 describes general information among different insurances. Patients with no insurance or commercial coverage tended to be younger with less chronic disease conditions, less likely homeless, and less likely to have a primary care physician (PCP) assigned in comparison to ones with other insurance coverages (Table 1).

**Table 1:** Study Patient General Characteristics

	No Insurance *N* = 99,026	Charity *N* = 41,427	Medicare *N* = 24,279	Medicaid *N* = 36,516	Commercial *N* = 26,379
Age --- year Median (IQR)	34 (26, 46)	44 (33, 53)	61 (50, 69)	37 (22,52)	38 (26,50)
Gender					
Male (*n*, %)	50,601 (51)	18,809 (45)	12,477 (51)	15,208 (42)	11,928 (45)
Mode of Arrival					
Healthcare Assisted (*n*, %)	19,713 (20)	9792 (24)	9150 (38)	11,663 (32)	6312 (24)
Homeless					
Yes (*n*, %)	4962 (5.0)	5651 (14)	2818 (12)	4708 (13)	363 (1.4)
Race					
Caucasian (*n*, %)	29,795 (30)	15,600 (38)	8852 (36)	10,631 (29)	8373 (32)
Non-Caucasian (*n*, %)	69,231 (70)	25,827 (62)	15,427 (64)	25,885 (71)	18,006 (68)
Level of acuity					
ESI-1,2,3 (*n*, %)	74,083 (75)	33,526 (81)	20,296 (84)	28,666 (79)	21,327 (81)
ESI-4,5 (*n*, %)	24,629 (25)	7803 (19)	3930 (16)	7760 (21)	4981 (19)
Medications Prescribed upon ED Discharge					
Median (IQR)	1 (0,2)	1 (0,2)	1 (0,2)	1 (0,2)	1 (0,2)
Mean (SD)	1.5 (1.4)	1.3 (1.5)	1.2 (1.9)	1.2 (1.5)	1.3 (1.5)
History of Chronic Diseases					
No (n, %)	57,065 (58)	18,299 (44)	8232 (34)	18,464 (51)	15,724 (60)
Yes (n, %)	41,961 (42)	23,128 (56)	16,047 (66)	18,052 (49)	10,655 (40)
Abnormal Vital Signs upon ED Discharge					
No (*n*, %)	84,181 (88)	35,136 (88)	20,076 (87)	28,705 (83)	22,386 (88)
Yes (*n*, %)	11,166 (12)	4723 (12)	2950 (13)	5835 (17)	2951 (12)
PCP Assigned					
No (*n*, %)	77,247 (78)	13,359 (32)	9409 (39)	16,923 (46)	16,028 (61)
Yes (*n*, %)	21,779 (22)	28,068 (68)	14,870 (61)	19,593 (54)	10,351 (39)

### A step-wise intervention improved patient clinic follow-up compliance

**Fig. 2:**
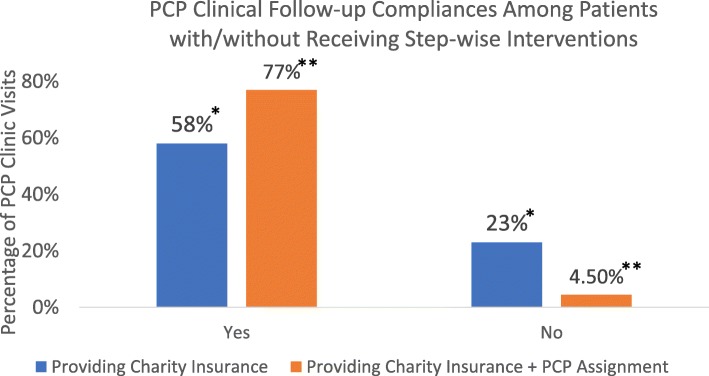
Outcome Comparison of Patients Receiving/Not Receiving Step-wise Interventions. *p< 0.001, **p< 0.001

**Fig. 3:**
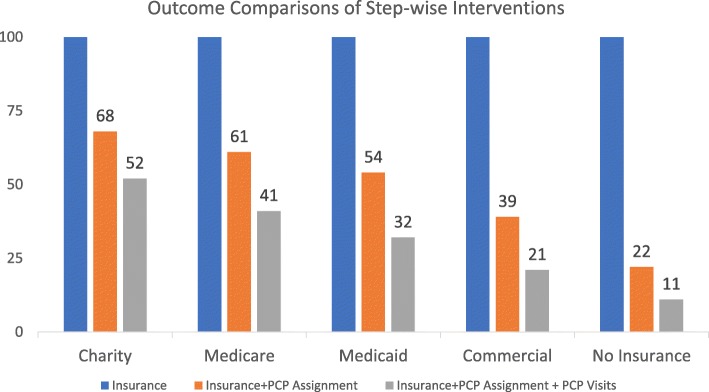
Comparison of Patient Involvement with Step-wise Interventions Relative to Different Insurance Coverages

Figure 2 shows that 58% (24,178/41,427) of patients who received charity insurance followed-up with a PCP clinic in comparison to only 23% (43,483/186,200) among patients who had no-charity insurance coverage (*p* < 0.001). Furthermore, 77% (21,695/28,068) of patients who received charity insurance with a dedicated PCP assignment completed clinic follow-ups in comparison to only 4.5% (5325/119,607) among patients who had no charity insurance and no dedicated PCP assignment (*p* < 0.001). Additionally, despite gradually decreased participant percentages with step-wise interventions, clinic follow-up compliance remained highest among patients who received charity insurance coverage and dedicated PCP assignment (Fig. 3).

### A step-wise intervention increased percentages of no-discharge-failure patients and reduced percentages of restricted-discharge-failure patients

Simultaneously, the percentage of patients with no discharge failure was also increased with the implementation of step-wise interventions. Increased no-discharge-failure rates were seen with the addition of dedicated PCP assignments and subsequent PCP visits. This was noted not only among patients with charity insurance but also ones without such benefits (Table 2). Meanwhile, restricted-discharge-failure rates were also reduced with the step-wise interventions specifically among patients receiving charity, Medicare, and Medicaid insurances (Table 2).

**Table 2:** Step-wise Interventions in related to Discharge Failure Outcomes

	No Discharge Failure	Restricted Discharge Failure	Broad/Uncertain Discharge Failure
Charity insurance (*N* = 41,427)	9078 (22)	7293 (18)	25,056 (60)
Charity insurance + PCP assignment (*N* = 28,068)	8159 (29)	4622 (16)	15,287 (54)
Charity insurance + PCP assignment + PCP visits (*N* = 21,695)	8159 (38)	3264 (15)	10,272 (47)
Medicare (*N* = 24,279)	3449 (14)	4531 (19)	16,299 (67)
Medicare + PCP assignment (*N* = 14,870)	3192 (21)	2504 (17)	9174 (62)
Medicare + PCP assignment + PCP visits (*N* = 10,057)	3192 (32)	1444 (14)	5421 (54)
Medicaid (*N* = 36,516)	4004 (11)	7485 (21)	25,027 (69)
Medicaid + PCP assignment (*N* = 19,593)	3544 (18)	3928 (20)	12,121 (62)
Medicaid + PCP assignment + PCP visits (*N* = 11,580)	3544 (31)	2095 (18)	5941 (51)
Commercial Insurance (*N* = 26,379)	3100 (18)	3388 (13)	19,891 (75)
Commercial insurance + PCP assignment (*N* = 10,351)	2783 (27)	1391 (13)	6177 (60)
Commercial insurance + PCP assignment + PCP visits (*N* = 5667)	2783 (49)	727 (13)	2157 (38)
No Insurance (*N* = 99,026)	3691 (4)	18,636 (19)	76,699 (77)
No insurance + PCP assignment (*N* = 21,779)	3223 (15)	4723 (22)	13,833 (64)
No insurance + PCP assignment + PCP visits (*N* = 10,854)	3223 (30)	2469 (23)	5162 (48)

### Urgent PCP clinic visits significantly reduced ED restricted discharge failures

When focused on patients who had clinic follow-up visits, we divided patients into 5 different groups based on the different urgencies of their PCP visits ranging from PCP visits ≤3 days from the index ED discharge to > 30 days. Urgent PCP clinic visits seemed to reduce patients with restricted discharge failures regardless of their insurance statuses (Table 3).

**Table 3:** Primary Care Physician Clinic Visits Associated with Discharge Failures

	No Discharge Failure	Restricted Discharge Failure	Broad/Uncertain Discharge Failure
Charity insurance + PCP assignment + PCP visits (*N* = 21,695)			
PCP visit ≤3 days from the index ED discharge (*N* = 1115)	584 (52)	10 (1)	521 (47)
PCP visit ≤7 days from the index ED discharge (*N* = 2592)	1302 (50)	73 (3)	1217 (47)
PCP visit ≤14 days from the index ED discharge (*N* = 4508)	2197 (49)	190 (4)	2121 (47)
PCP visit ≤30 days from the index ED discharge (*N* = 7732)	3536 (46)	534 (7)	3662 (47)
PCP visit > 30 days from the index ED discharge (*N* = 13,963)	4623 (33)	2730 (20)	6610 (47)
Medicare + PCP assignment + PCP visits (*N* = 10,057)			
PCP visit ≤3 days from the index ED discharge (*N* = 499)	235 (47)	12 (2)	252 (51)
PCP visit ≤7 days from the index ED discharge (*N* = 1173)	545 (46)	41 (4)	587 (50)
PCP visit ≤14 days from the index ED discharge (*N* = 2158)	957 (44)	110 (5)	1091 (51)
PCP visit ≤30 days from the index ED discharge (*N* = 3740)	1546 (41)	257 (7)	1937 (52)
PCP visit > 30 days from the index ED discharge (*N* = 6317)	1646 (26)	1187 (19)	3484 (55)
Medicaid + PCP assignment + PCP visits (*N* = 11,580)			
PCP visit ≤3 days from the index ED discharge (*N* = 474)	231 (49)	10 (2)	233 (49)
PCP visit ≤7 days from the index ED discharge (*N* = 1097)	501 (46)	32 (3)	564 (51)
PCP visit ≤14 days from the index ED discharge (*N* = 1955)	869 (44)	107 (5)	979 (50)
PCP visit ≤30 days from the index ED discharge (*N* = 3462)	1377 (40)	302 (9)	1783 (52)
PCP visit > 30 days from the index ED discharge (*N* = 8118)	2167 (27)	1793 (22)	4158 (51)
Commercial insurance + PCP assignment + PCP visits (*N* = 5667)			
PCP visit ≤3 days from the index ED discharge (*N* = 259)	167 (64)	5 (2)	87 (34)
PCP visit ≤7 days from the index ED discharge (*N* = 629)	375 (60)	19 (3)	235 (37)
PCP visit ≤14 days from the index ED discharge (*N* = 1070)	628 (59)	43 (4)	399 (37)
PCP visit ≤30 days from the index ED discharge (*N* = 1779)	1037 (58)	69 (4)	673 (38)
PCP visit > 30 days from the index ED discharge (*N* = 3888)	1746 (45)	658 (17)	1484 (38)
No insurance + PCP assignment + PCP visits (*N* = 10,854)			
PCP visit ≤3 days from the index ED discharge (*N* = 214)	128 (60)	2 (1)	84 (39)
PCP visit ≤7 days from the index ED discharge (*N* = 449)	265 (59)	8 (2)	176 (39)
PCP visit ≤14 days from the index ED discharge (*N* = 847)	451 (53)	48 (6)	348 (41)
PCP visit ≤30 days from the index ED discharge (*N* = 1473)	722 (49)	111 (8)	640 (43)
PCP visit > 30 days from the index ED discharge (*N* = 9381)	2501 (27)	2358 (25)	4522 (48)

### Step-wise interventions associated with clinic compliance and ED restricted-discharge failures using multivariate logistic regression with intervention-interaction analysis

To avoid the potential confounders, a multivariate logistic regression was performed, adjusted with all potential independent variables. When using patients with no insurance and no PCP assignment as the reference, our study found that insurance coverage and PCP assignment are tightly associated with patient clinic follow-up visits at both individual and combination levels (Table 4). The same model was used to determine the association between interventions and ED restricted discharge failure. We found that patients with either charity or commercial insurance and who completed PCP visits were associated with less ED restricted discharge failure if analyzed individually (Table 5). However, when three interventions were analyzed with interactions to further predict restricted ED discharge failures, insurance coverage with charity, Medicare, or Medicaid itself did not seem to reduce ED discharge failure. However, such outcome benefits occurred with the combinations of step-wise interventions even after adjusted with all the confounders (Table 5).

**Table 4:** Adjusted Odds Ratios of Step-wise Interventions Predictive of Patient Clinical Compliance in a Multivariate Logistic Regression Model with Intervention Interaction Analysis

	Adjusted Odds Ratios	(95% Confidence Interval)
Age	1.02	1.02–1.02
Race		
Caucasian	Reference	Reference
Non-Caucasian	1.04	1.02–1.07
Homeless		
No	Reference	Reference
Yes	1.11	1.06–1.16
Vital Signs upon ED Discharge		
Normal	Reference	Reference
Abnormal	1.03	0.99–1.07
Patient Level of Acuity		
High Acuity level (ESI1–3)	Reference	Reference
Low Acuity Level (ESI4–5)	0.98	0.95–1.02
Patient with Chronic Diseases		
No	Reference	Reference
Yes	1.07	1.04–1.09
Insurance type		
No insurance	Reference	Reference
Charity	4.51	4.35–4.67
Medicare	2.23	2.13–2.33
Medicaid	2.01	1.94–2.08
Commercial	1.38	1.32–1.44
PCP Assignment		
No	Reference	Reference
Yes	20	19–20
Step-wise Interventions with interactions		
No Insurance + No PCP-Assignment	Reference	Reference
Charity Insurance	10	10–11
Charity Insurance + PCP Assignment	148	139–157
Medicare Insurance	6.76	6.24–7.32
Medicare Insurance + PCP Assignment	67	63–92
Medicaid Insurance	5.09	4.72–5.50
Medicaid Insurance + PCP Assignment	66	62–70
Commercial Insurance	2.06	1.87–2.27
Commercial Insurance + PCP Assignment	52	49–56

**Table 5:** Adjusted Odds Ratios of Step-wise Interventions Predictive of ED Restricted-Discharge Failure in a Multivariate Logistic Regression Model with Intervention Interaction Analysis

	Adjusted Odds Ratios	(95% Confidence Interval)
Age	1.06	1.04–1.08
Race		
Caucasian	Reference	Reference
Non-Caucasian	1.06	1.04–1.09
Homeless		
No	Reference	Reference
Yes	2.03	1.96–2.10
Vital Signs upon ED Discharge		
Normal	Reference	Reference
Abnormal	1.21	1.17–1.26
Patient Level of Acuity		
High Acuity level (ESI1–3)	Reference	Reference
Low Acuity Level (ESI4–5)	1.50	1.46–1.54
Patient with Chronic Diseases		
No	Reference	Reference
Yes	1.17	1.14–1.20
Insurance type		
No insurance	Reference	Reference
Charity	0.91	0.88–0.95
Medicare	0.98	0.94–1.02
Medicaid	1.06	1.03–1.10
Commercial	0.67	0.64–0.69
PCP Assignment		
No	Reference	Reference
Yes	1.13	1.09–1.16
PCP Visit		
No	Reference	Reference
Yes	0.78	0.75–0.80
Step-wise Interventions with interactions		
No Insurance + No PCP-Assignment + No PCP Visits	Reference	Reference
Charity Insurance	1.17	1.11–1.24
Charity Insurance + PCP Assignment	1.15	1.07–1.22
Charity Insurance + PCP Assignment + PCP Visits	0.67	0.60–0.76
Medicare Insurance	1.43	1.27–1.60
Medicare Insurance + PCP Assignment	1.22	1.14–1.32
Medicare Insurance + PCP Assignment + PCP Visits	0.73	0.69–0.78
Medicaid Insurance	1.18	1.12–1.23
Medicaid Insurance + PCP Assignment	0.93	0.81–1.07
Medicaid Insurance + PCP Assignment + PCP Visits	0.92	0.87–0.97
Commercial Insurance	0.69	0.65–0.73
Commercial Insurance + PCP Assignment	0.66	0.52–0.84
Commercial Insurance + PCP Assignment + PCP Visits	0.68	0.63–0.74

## Discussion

Timely arrangement of post-ED follow-up is critical to ensure patient safety, monitor patient disease progression, and adjust management regimens properly [2, 18]. However, increased post-ED follow-up clinic visit rates do not simultaneously reduce inappropriate ED utilization or ED returns [11, 19]. In this study, we found approximately 30% of patients had PCP visits, while 18% of this cohort had restricted ED discharge failures. In addition, the more urgent the PCP clinic visits (e.g., within 30 days from index ED discharge), the lower the rate of restricted ED discharge failure. This also indicates such step-wise interventions could significantly improve patient PCP clinic follow-up compliance and reduce inappropriate ED return/utilization rates, but at different levels. Such interventions tend to increase patient clinic follow-up more significantly than reducing ED discharge failures. We realized individual interventions might not improve ED discharge failures and also noted that it may be pragmatically challenging to just choosing effective interventions without implementing upstream interventions. As a result, we followed this common step-wise implementation pathway, analyzed intervention-interactions at each level, and evaluated the value of each intervention in a more pragmatic manner.

Our findings emphasize the importance of implementing such interventions and recognize the differences between patient follow-up clinic compliance and ED discharge failure. Furthermore, our study determines that current interventions with urgent PCP visits might not only improve patient clinic follow-up compliance but also reduce inappropriate ED utilization. Our results add value to the current literature by introducing results of a large systematic outcome study with pragmatic step-wise interventions which has not been reported to date.

This study has several strengths: 1) a large sample size with application to diverse concepts of discharge failure models; 2) systematic evaluation of patient follow-up compliance using pragmatic step-wise interventions; and 3) broad comparative outcomes between different patient populations with greater potential for translation to the general population (e.g., patients with different insurance coverage, patients with different time intervals from index ED discharge to subsequent PCP clinic visits, etc.).

There are diverse concepts of ED discharge failure models reported in the literature (e.g., 72-h ED returns, ED/hospital readmission within 30 days, inappropriate ED utilization/high ED utilizers, etc.) [8, 9, 20, 21]. Here, we simplified and introduced two discharge failure models having either broad/uncertain discharge failure potential or short-term (< 30 days) restricted discharge failure. Given the uncertainty of ED returns and poor adherence of patient clinic follow-up, we believe that such restricted discharge failure model can minimize potential bias. All the differentiations used in this study seemed important to narrow down the “true” discharge failure cohort.

Different findings are reported among the current literature regarding the association between patient PCP clinic follow-up compliance and ED returns/revisits [11, 19]. Some studies focusing on asthma patients revealed the value of urgent PCP follow-ups [22] and their impact on decreased ED returns [23], while others yielded opposite findings [24, 25]. Patients with Medicare/Medicaid tended to have more ED returns regardless of PCP clinic visits [26, 27]. Other studies showed homeless patients and those with high psychosocial risks might breach the benefit of such PCP referrals and are recognized as high ED utilizers [11, 28, 29]. Such mixed findings of previous studies indicate that PCP referral might be beneficial only among certain patient populations. In this study, we considered all ED discharged patients as a cohort and further sub-grouped patients with different insurance coverage. Our results are quite similar to the findings from previous literature [30, 31]. Future research should be focused on population-based studies related to different ED discharge failure models.

Our study has limitations. First, given the nature of single-center, retrospective data analysis studies in general, limited and potential incorrect information, and missing data, potential patient population selection bias cannot be avoided. Second, differentiating ED discharge failures into restricted and broad/uncertain might not be accurate since we were not able to accurately differentiate between patients with certain uncertainties. Additionally, we only enrolled patients seeking healthcare at the study hospital/ED. We are unable to know the percentage of patients who had appointments in other hospitals or followed-up at PCP clinics outside of the study hospital system. This could further deviate the accuracy of study findings. Third, though all our patients who had follow-up appointments assigned will receive a phone call prior to their appointment, we were unable to know the success rate of these phone calls being answered. Therefore, we did not consider phone call interview an effective intervention and did not include it in our final analysis. Fourth, we analyzed data based on each ED discharge and some individual patients might have logged multiple ED encounters during the study period. This might impact final results if this cohort of patients were high utilizers of ED services. Therefore, a future multi-center prospective population-based study is warranted for further validation.

## Conclusion

In summary, higher ED discharge failures occurred in the study cohort due to the diverse concepts of discharge failure models. Interventions such as providing hospital-based charity insurance coverage, assigning a dedicated PCP, and providing a follow-up phone reminder in a step-wise fashion increased patient PCP clinic visit compliance while simultaneously reducing ED restricted discharge failures. More specifically, urgent referral to PCP clinics within 30 days seemed to improve ED discharge failure rates.

## Additional file

Additional file 1: Appendix: A detail definition of different outcome measurements. (DOCX 14 kb)

## Data Availability

The data that support the findings of this study are available upon requesting to an authorized person via email (research@jpshealth.org, Dr. Melissa Acosta).

## Abbreviation

AHRQ: Agency for Healthcare Research and Quality
AMA: Against Medical Advice
CCI: Chronic Condition Indicator
ED: Emergency Department
EM: Emergency medicine
EMR: Electronic medical record
ESI: Emergency severity index
FPIL: Federal poverty income level
HCUP: Healthcare cost and utilization project
ICD-10-CM: International Classification of Diseases Tenth Edition, Clinical Modification
IQR: Interquartile range
LMSW: Licensed Master Social Workers
N: Number
NYUA: New York University Algorithm
PCP: Primary care physician
SD: Standard deviation
TX: Texas
US: United States

## References

[1] Misky GJ, Wald HL, Coleman EA (2010). Post-hospitalization transitions: examining the effects of timing of primary care provider follow-up. J Hosp Med.

[2] Schull MJ (2014). Making aftercare more than an afterthought: patient follow-up after emergency department discharge in Ontario. Healthc Q.

[3] Koehler BE, Richter KM, Youngblood L, Cohen BA, Prengler ID, Cheng D (2009). Reduction of 30-day postdischarge hospital readmission or emergency department (ED) visit rates in high-risk elderly medical patients through delivery of a targeted care bundle. J Hosp Med.

[4] Wong FK, Chow S, Chang K, Lee A, Liu J (2004). Effects of nurse follow-up on emergency room revisits: a randomized controlled trial. Soc Sci Med.

[5] Horwitz SM, Busch SH, Balestracci KM, Ellingson KD, Rawlings J (2005). Intensive intervention improves primary care follow-up for uninsured emergency department patients. Acad Emerg Med.

[6] McCusker J, Dendukuri N, Tousignant P, Verdon J, Poulin de CL, Belzile E (2003). Rapid two-stage emergency department intervention for seniors: impact on continuity of care. Acad Emerg Med.

[7] Atzema CL, Maclagan LC (2017). The transition of care between emergency department and primary care: a scoping study. Acad Emerg Med.

[8] Pham JC, Kirsch TD, Hill PM, DeRuggerio K, Hoffmann B (2011). Seventy-two-hour returns may not be a good indicator of safety in the emergency department: a national study. Acad Emerg Med.

[9] Magnusson AR, Hedges JR, Vanko M, McCarten K, Moorhead JC (1993). Follow-up compliance after emergency department evaluation. Ann Emerg Med.

[10] Boudreaux ED, Clark S, Camargo CA (2000). Telephone follow-up after the emergency department visit: experience with acute asthma. On behalf of the MARC Investigators. Ann Emerg Med.

[11] Wang H, Nejtek VA, Zieger D, Robinson RD, Schrader CD, Phariss C (2015). The role of charity care and primary care physician assignment on ED use in homeless patients. Am J Emerg Med.

[12] McCarthy ML, Hirshon JM, Ruggles RL, Docimo AB, Welinsky M, Bessman ES (2002). Referral of medically uninsured emergency department patients to primary care. Acad Emerg Med.

[13] Naderi S, Barnett B, Hoffman RS, Dalipi R, Houdek L, Alagappan K (2012). Factors associated with failure to follow-up at a medical clinic after an ED visit. Am J Emerg Med.

[14] Sharp B, Singal B, Pulia M, Fowler J, Simmons S (2015). You've got mail ... And need follow-up: the effect and patient perception of e-mail follow-up reminders after emergency department discharge. Acad Emerg Med.

[15] Hegney D, Buikstra E, Chamberlain C, March J, McKay M, Cope G (2006). Nurse discharge planning in the emergency department: a Toowoomba, Australia, study. J Clin Nurs.

[16] Ballard DW, Price M, Fung V, Brand R, Reed ME, Fireman B (2010). Validation of an algorithm for categorizing the severity of hospital emergency department visits. Med Care.

[17] Agency for Healthcare Research and Quality (2018). Beta Chronic Condition Indicator (CCI) for ICD-10-CM.

[18] Schectman JM, Schorling JB, Voss JD (2008). Appointment adherence and disparities in outcomes among patients with diabetes. J Gen Intern Med.

[19] Moskovitz JB, Ginsberg Z (2015). Emergency department Bouncebacks: is lack of primary care access the primary cause?. J Emerg Med.

[20] Klinkenberg WD, Calsyn RJ (1997). The moderating effects of race on return visits to the psychiatric emergency room. Psychiatr Serv.

[21] Khan NU, Razzak JA, Saleem AF, Khan UR, Mir MU, Aashiq B (2011). Unplanned return visit to emergency department: a descriptive study from a tertiary care hospital in a low-income country. Eur J Emerg Med.

[22] Rizza P, Bianco A, Pavia M, Angelillo IF (2007). Preventable hospitalization and access to primary health care in an area of southern Italy. BMC Health Serv Res.

[23] Smith SR, Wakefield DB, Cloutier MM (2007). Relationship between pediatric primary provider visits and acute asthma ED visits. Pediatr Pulmonol.

[24] Cabana MD, Bruckman D, Bratton SL, Kemper AR, Clark NM (2003). Association between outpatient follow-up and pediatric emergency department asthma visits. J Asthma.

[25] Nelson KA, Garbutt JM, Wallendorf MJ, Trinkaus KM, Strunk RC (2014). Primary care visits for asthma monitoring over time and association with acute asthma visits for urban Medicaid-insured children. J Asthma.

[26] Taubman SL, Allen HL, Wright BJ, Baicker K, Finkelstein AN (2014). Medicaid increases emergency-department use: evidence from Oregon's health insurance experiment. Science.

[27] Hunold KM, Richmond NL, Waller AE, Cutchin MP, Voss PR, Platts-Mills TF (2014). Primary care availability and emergency department use by older adults: a population-based analysis. J Am Geriatr Soc.

[28] Han B, Wells BL (2003). Inappropriate emergency department visits and use of the health Care for the Homeless Program services by homeless adults in the northeastern United States. J Public Health Manag Pract.

[29] Hategan A, Tisi D, Abdurrahman M, Bourgeois JA (2016). Geriatric homelessness: association with emergency department utilization. Can Geriatr J.

[30] Pukurdpol P, Wiler JL, Hsia RY, Ginde AA (2014). Association of Medicare and Medicaid insurance with increasing primary care-treatable emergency department visits in the United States. Acad Emerg Med.

[31] Katz DA, McCoy KD, Vaughan-Sarrazin MS (2015). Does greater continuity of veterans administration primary care reduce emergency department visits and hospitalization in older veterans?. J Am Geriatr Soc.

