# Supplementary material for: Common step-wise interventions improved primary care clinic visits and reduced emergency department discharge failures: a large-scale retrospective observational study
Source: BMC Health Serv Res. 2019 Jul 4;19:451. doi: 10.1186/s12913-019-4300-1 (PMC6610992; doi:10.1186/s12913-019-4300-1)
Supplement: Supplementary file 1 — Appendix: A detail definition of different outcome measurements. (DOCX 14 kb) [file 12913_2019_4300_MOESM1_ESM.docx]

Additional File: A detail definition of different outcome measurements

| Different Outcome Measurements | Detail Definitions |
| --- | --- |
| Patient follow-up compliance | Patients who visited the primary care physician clinic after the index ED discharge regardless of ED revisits/returns |
| Emergency Department Discharge Failures |  |
| Broad/Uncertain ED discharge failures | Need to meet one of the following criteria:  1) patients who had neither subsequent ED nor clinic visits;  2) patients had appropriate or unclassified ED returns but did not have any PCP follow-ups from the index ED discharge;  3) patients returned to the ED appropriately or unclassified prior to their clinic follow-ups;  4) patients returned to the ED after PCP visits and their ED returns were inappropriate or unclassified |
| Restricted ED discharge failures |  |
| Restricted ED discharge failures within 3 days  Restricted ED discharge failures within 7 days  Restricted ED discharge failures within 14 days  Restricted ED discharge failures within 30 days | Need to meet all the following criteria:   1. patients revisited the ED prior to their scheduled clinic follow-up visit or patients revisited the ED with no scheduled clinic follow-up since index ED visit, 2. patients with a return ED visit that resulted in ED discharge and was considered inappropriate ED utilization (i.e., non-emergent, avoidable) determined by NYUA.   Special analysis was rendered if patient ED discharge failures occurred within 3, 7, 14, and 30 days from the index ED discharge. |
